# Association between self-reported eye conditions in patients at rural federally qualified health centers and vision-targeted health-related quality of life: the AL-SIGHT study

**DOI:** 10.3389/fmed.2025.1498413

**Published:** 2025-01-15

**Authors:** Thomas A. Swain, Gerald McGwin, Lindsay A. Rhodes, Christopher A. Girkin, Cynthia Owsley

**Affiliations:** ^1^Department of Ophthalmology and Visual Sciences, Heersink School of Medicine, University of Alabama at Birmingham, Birmingham, AL, United States; ^2^Department of Epidemiology, School of Public Health, University of Alabama at Birmingham, Birmingham, AL, United States

**Keywords:** federally qualified health center, glaucoma, rural health, quality of life, National Eye Institute Visual Function Questionnaire-9

## Abstract

**Background:**

Federally Qualified Health Centers (FQHCs) are safety-net primary health care clinics in the US serving medically underserved areas and populations. We administered the National Eye Institute Visual Function Questionnaire – 9 (VFQ-9), a vision-targeted, health-related quality of life questionnaire, to patients in 3 FQHCs in rural Alabama at risk for glaucoma. We examined demographic factors and self-reported eye conditions associated with VFQ-9 scores.

**Methods:**

The VFQ-9 (score range 0–100) was administered to patients at-risk for glaucoma including African Americans or Hispanics ≥40 years, white persons ≥50 years, persons with diabetes ≥18 years, ≥18 years with glaucoma or glaucoma suspect, and/or ≥ 18 years with a family history of glaucoma. Demographic variables were collected -- age, gender, race/ethnicity, employment, marital status, health insurance, education, and driving status. Patients reported the presence of eye conditions including glaucoma and many other eye conditions. Stepwise linear regression modeled which variables accounted for the greatest variance of the VFQ-9 score.

**Results:**

Composite VFQ-9 scores averaged 82.4. The best fitting model for VFQ-9 scores included being a driver, insurance type/status, self-reported glaucoma or glaucoma suspect, blurry vision, and double vision.

**Conclusion:**

Patients at-risk for glaucoma seeking care at FQHCs in rural Alabama have moderate impairment in quality of life as assessed by the VFQ-9. Factors negatively influencing scores are self-reported glaucoma or glaucoma suspect, blurry vision, double vision, not being a driver, and having no health insurance. The VFQ-9 is a good candidate as a vision-targeted quality of life outcome for eye health interventions at rural FQHCs in those with glaucoma.

## 1 Introduction

Federally Qualified Health Centers (FQHCs) are safety-net primary health care clinics in the United States designed to serve medically underserved areas and populations in both rural and urban regions. They provide services regardless of the patient’s ability to pay, using a sliding-scale fee based on the ability to pay. While they provide primary medical care in many health domains, one domain that is not well addressed by FQHCs is eye care. A recent report by the National Academies of Science, Engineering and Medicine indicated that primary eye care services (through optometrists or ophthalmologists) are rarely available at FQHCs; the report provided an estimate that less than 3% of FQHC patients actually receive vision care services at FQHCs, representing 0.89% of FQHC clinic visits (1).

An important question is whether telemedicine services could address this significant need in rural areas by combining this technology with FQHC primary care services to optimize a practical and cost-effective screening strategy to identify eye conditions. In telemedicine, the patient’s screening information is electronically sent from the FQHC to a remote ophthalmologist for diagnosis who makes a recommendation for follow-up referral as needed. In addition, with the emergence of non-invasive ocular imaging tools with higher diagnostic reliability, electronic transferability, and ease of use, telemedicine has shown promise as compared to in-person diagnosis of diabetic retinopathy, age-related macular degeneration, and glaucoma (2, 3). The COVID-19 pandemic heightened the need for telemedicine, and thus the rise in its use since the pandemic (4).

Recently we conducted a telemedicine screening program based in three FQHCs located in rural Alabama areas called Alabama Screening and Intervention for Glaucoma and Eye Health through Telemedicine (AL-SIGHT) (5). We focused on patients who were at-risk for glaucoma associated disease (GAD), which includes glaucoma, ocular hypertension, and glaucoma suspect. The at-risk population for GAD includes White persons ≥50 years of age, African American and Hispanic people ≥40 years of age, and people with a family history of GAD and/or diabetes (6). Before we performed the vision screening, we queried patients’ demographics and own awareness of any eye conditions they had using a self-report structured questionnaire. In addition, we also administered a short-form version of a commonly used vision-targeted, health-related quality of life questionnaire. The purpose of this analysis was to examine the association between vision-targeted quality of life and patient demographics and self-awareness about eye conditions at these FQHCs.

## 2 Methods

This study was approved by the Institutional Review Board of the University of Alabama at Birmingham and adhered to the tenets of the Declaration of Helsinki. Participants provided written informed consent after the nature and purpose of the study was explained.

### 2.1 Data source

The three FQHC sites were part of the Cahaba Medical Care Foundation, an Alabama-based FQHC, and are located in rural areas. These FQHCs provide health care for approximately 16,000 patients per year, over half of whom are African American. Fifty-five percent of their patient population have Medicaid or Medicare, 25% have private insurance and 20% are uninsured. The study clinics are in the following Alabama towns: Centreville (Bibb County), Maplesville (Chilton County), and Marion (Perry County). This region of Alabama borders or is part of the region known as the Black Belt named for its rich black soil that supported cotton agriculture for many decades. In the 19th century, the agricultural workers were enslaved African Americans. The Black Belt consists of 9 of 10 of the poorest counties in the state. Poverty is directly linked to health disparities in the Black Belt (7). Today this region’s population is over 50% African American.

Patients presenting at these clinics were eligible to participate in the study if they had one or more risk factors for GAD and volunteered to participate: (1) African American or Hispanic ≥40 years of age; (2) White persons ≥50 years of age; (3) anyone ≥18 years of age with diabetes, (4) anyone ≥18 years of age with a GAD; and (5) anyone ≥18 years of age with a family history of glaucoma. All participants spoke and understood English. Although the inclusion criteria are focused on patients at-risk for glaucoma, we asked about whether they had many eye conditions as described below. All structured questionnaires were interviewer-administered.

### 2.2 Protocol

Questionnaires addressed the following: birthdate, sex, race/ethnicity, employment status, marital status, health insurance status, educational level attained, and the transportation used to attend the screening. We also asked participants whether they had any of the following eye conditions before they learned the results of their vision screening: glaucoma or glaucoma suspect, refractive error (defined as near-sightedness, far-sightedness, astigmatism, presbyopia), dry eye, blurry vision, double vision, cataract, high eye pressure, diabetic retinopathy, age-related macular degeneration, and floaters. Response options were yes versus no.

We also administered the short-form of the National Eye Institute Visual Function Questionnaire – 25 (NEI VFQ-25), a vision-targeted health-related quality of life questionnaire (8, 9). The National Eye Institute Visual Function Questionnaire - 9 (NEI VFQ-9) (10) has demonstrated excellent reliability and validity (Table 1). Content domains addressed are general vision, near and distance activities, mental health, role difficulties, driving, and peripheral vision. Values ranging from 0 to 100 were assigned to response options for each question as indicated by the NEI VFQ-25 scoring methods (11). Higher values correspond to better functioning and quality of life. Each question comprised a single subscale except near vision, for which the mean of 3 items made the subscale. The composite score was an average of all subscales. If persons were no longer driving or never drove, the composite was the mean of the other 6 subscales.

**Table 1:** Items in the National Eye Institute Visual Function Questionnaire – 9 (NEI VFQ-9) (10).

Item
Q1. At the present time, would you say your eyesight (with glasses or contact lenses, if you wear them) is:
(1) Excellent, (2) good, (3) fair, (4) poor, (5) very poor, or (6) are you completely blind?
Q2. How much of the time do you worry about your eyesight?
(1) None of the time, (2) a little of time, (3) some of the time, (4) most of the time, or (5) all of the time.
Q3. How much difficulty do you have reading ordinary print in newspapers?
(1) No difficulty at all, (2) a little difficulty, (3) moderate difficulty, (4) extreme difficulty, (5) stopped doing because of your eyesight, or (6) stopped doing this for other reasons or not interested in doing this.
Q4. How much difficulty do you have doing work or hobbies that require you to see well up close, such as cooking, sewing, fixing things around the house, or using hand tools?
(1) No difficulty at all, (2) a little difficulty, (3) moderate difficulty, (4) extreme difficulty, (5) stopped doing because of your eyesight, or (6) stopped doing this for other reasons or not interested in doing this.
Q5. Because of your eyesight, how much difficulty do you have going down steps, stairs, or curbs in dim light or at night?
(1) No difficulty at all, (2) a little difficulty, (3) moderate difficulty, (4) extreme difficulty, (5) stopped doing because of your eyesight, or (6) stopped doing this for other reasons or not interested in doing this.
Q6. How much difficulty do you have driving during the daytime in familiar places?
(1) No difficulty at all, (2) a little difficulty, (3) moderate difficulty, (4) extreme difficulty, stopped doing because of your eyesight, or (5) stopped doing this for other reasons or not interested in doing this.
Q7. Are you limited in how long you can walk or do other activities such as housework, child care, school, or community activities because of your vision?
(1) All of the time, (2) most of the time, (3) some of the time, (4) a little of time, or (5) none of the time.
Q8. Because of your eyesight, how much difficulty do you have noticing objects off to the side while you are walking along?
(1) No difficulty at all, (2) a little difficulty, (3) moderate difficulty, (4) extreme difficulty, (5) stopped doing because of your eyesight, or (6) stopped doing this for other reasons or not interested in doing this.
Q9. Because of your eyesight, how much difficulty do you have finding something on a crowded shelf?
(1) No difficulty at all, (2) a little difficulty, (3) moderate difficulty, (4) extreme difficulty, (5) stopped doing because of your eyesight, or (6) stopped doing this for other reasons or not interested in doing this.

Response options are listed below each item.

### 2.3 Statistical analysis

Mean (standard deviation) and number (percent) were used to summarize continuous and categorical variables, respectively. The VFQ-9 composite score was compared by participant characteristics and self-reported eye conditions using linear regression adjusting for age (except the comparison by age category). The level of significance was *p* ≤ 0.05 (two-sided). To model which variables accounted for the greatest variance of the VFQ-9 score, meaning the participant factors which statistically explained the score value, stepwise linear regression was completed for demographics and other characteristics, self-reported eye conditions, and both of these data groupings combined. The *p*-value was set to 0.05 for model entry and 0.01 to stay in the model. Corrected Akaike information criterion (AICC) and adjusted R^2^ values were examined in the modeling process. Models with higher adjusted R^2^ have greater accuracy and explained variance. AICCs are relative indices used in model selection and reflect goodness of fit, with lower values indicative of a more parsimonious model. All analyses were conducted in SAS, version 9.4 (SAS Institute, Cary, NC).

## 3 Results

A total of 500 persons enrolled and comprised the analysis sample across the three FQHC clinics. Over 90% of participants were ≥ 40 years old, with 2/3 women (Table 2). African Americans and white persons were approximately equally represented. Those who were unemployed represented 38.6% of the sample. Over half were married or had a domestic partner. With respect to health insurance, 45.6% had Medicaid or Medicare, with 18.2% with no health insurance; 23.9% did not complete high school.

**Table 2:** Demographic characteristics of the sample (*N* = 500).

	*n* (%)
Age, years	
18–39	43 (8.6)
40–59	247 (49.4)
≥ 60	210 (42.0)
Gender	
Men	178 (35.6)
Women	322 (64.4)
Race	
African American	228 (45.6)
White persons	258 (51.6)
Other^1^	14 (2.8)
Employment	
Employed full- or part-time	171 (34.2)
Retired	136 (27.2)
Unemployed or unable to work	193 (38.6)
Marital status	
Married or domestic partnership	255 (51.0)
Divorced, separated, or single	190 (38.0)
Widowed	55 (11.0)
Health insurance	
Medicaid	101 (20.2)
Medicare	127 (25.4)
Private insurance	181 (36.2)
No insurance	91 (18.2)
Education^2^	
Less than high school	119 (23.9)
High school graduate or more	380 (76.2)
Transportation	
Drove themselves to screening	371 (74.2)
Someone else drove them, public transportation, or walked^3^	129 (25.8)

^1^Other included American Indian or Alaskan Native (*N* = 1) and multiracial (*N* = 13).

^2One person declined to provide education information.^

^3119 participants had someone else drive them, 2 used public transportation, and 8 walked.^

**Figure 1:**
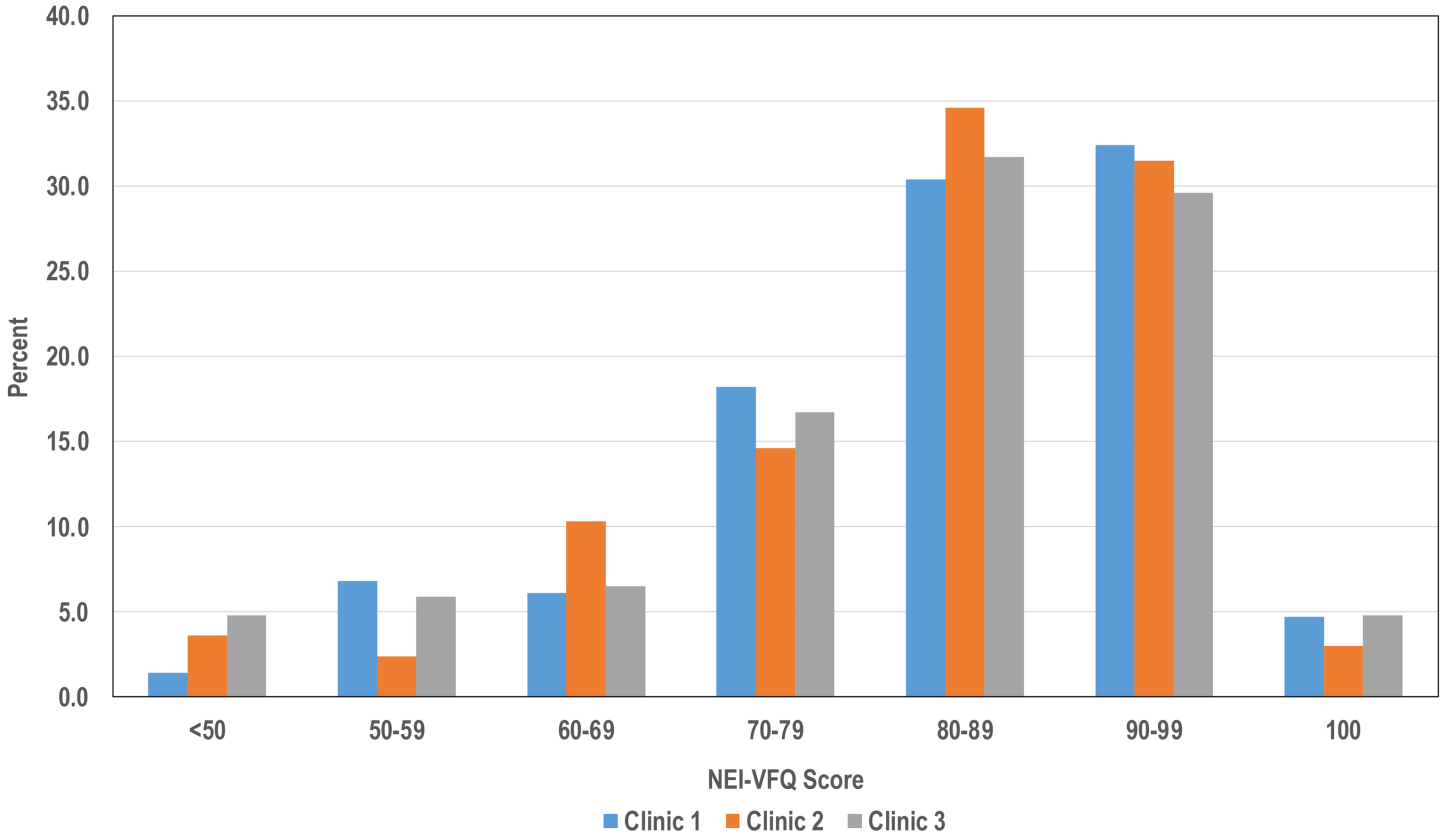
The distribution of VFQ-9 composite scores (expressed as a percentage) stratified by the three FQHC clinics.

NEI VFQ-9 scores differed with respect to several demographic characteristics (Table 3) adjusting each variable for all the other variables in Table 3. Women had lower scores than men; however, this difference was modest at 3 points. Several characteristics led to a more substantial difference in scores. Those who were unemployed or unable to work had on average 5 points lower than those who worked or were retired; those who had no health insurance had on average 3–4 points lower than the other insurance categories. Persons who had someone else drive them, walked, or used public transportation to attend the screening visit had scores 5 points lower than those who drove themselves. Marital status had no impact on VFQ-9 scores. Figure 1 shows the distribution of VFQ-9 composite scores (expressed as a percentage) stratified by the three FQHC clinic.

**Table 3:** Visual Function Questionnaire – 9 (VFQ-9) total score stratified by demographic characteristics including adjustments for other demographic variables.

	*n* (%)	Mean VFQ-9 total score (standard deviation)	*p*-value	Adjusted mean VFQ-9 total score	Adjusted *p*-value
Age, years			0.005		0.9463
18–39	43 (8.6)	84.3 (14.5)		80.1	
40–59	247 (49.4)	80.4 (14.2)		79.4	
≥ 60	210 (42.0)	84.4 (12.9)		79.5	
			Age-adjusted *p*-value		
Gender	178 (35.6)		0.005		0.0136
Men	322 (64.4)	84.8 (12.7)		81.2	
Women		81.1 (14.2)		78.1	
Race			0.013		0.1749
African American	228 (45.6)	81.2 (13.7)		81.6	
White persons	258 (51.6)	83.9 (13.0)		82.0	
Other	14 (2.8)	75.1 (23.4)		75.4	
Employment			<0.0001		0.0068
Employed full- or part-time	171 (34.2)	85.7 (11.6)		81.4	
Retired	136 (27.2)	85.0 (12.4)		80.1	
Unemployed or unable to work	193 (38.6)	77.8 (15.2)		76.0	
Marital status			0.606		0.7308
Married or domestic partnership	255 (51.0)	82.9 (13.3)		79.0	
Divorced, separated, or single	190 (38.0)	81.5 (14.2)		79.5	
Widowed	55 (11.0)	83.5 (14.6)		80.5	
Health insurance			<0.0001		0.0125
Medicaid	101 (20.2)	78.9 (15.4)		79.4	
Medicare	127 (25.4)	85.0 (10.7)		82.5	
Private insurance	181 (36.2)	85.1 (12.2)		80.7	
No insurance	91 (18.2)	77.5 (16.4)		76.0	
Education			0.003	78.7	0.1618
Less than high school	119 (23.9)	79.3 (14.5)		80.7	
High school graduate or more	380 (76.2)	83.4 (13.4)			
Transportation			<0.0001		<0.0001
Drove themselves to screening	371 (74.2)	84.7 (11.8)		83.0	
Someone else drove them, public transportation, or walked	129 (25.8)	75.9 (16.7)		78.7	

The largest impacts on VFQ-9 scores in terms of self-reported eye conditions were for blurry vision, double vision, and diabetic retinopathy, with these conditions lowering the score by about 7 to 13 points depending on the condition (Table 4). Other conditions had more modest impacts in lowering scores, including glaucoma or glaucoma suspect status, dry eye, and floaters, lowering scores by approximately 3 points. Self-reported high eye pressure, cataract, and refractive error had no impact on VFQ-9 scores.

**Table 4:** Visual Function Questionnaire – 9 (VFQ-9) total score stratified by self-reported eye conditions.

	*n* (%)	Mean VFQ-9 total score (standard deviation)	Age-adjusted *p*-value
Glaucoma or glaucoma suspect			0.024
Yes	70 (14.0)	79.0 (13.3)	
No	430 (86.0)	83.0 (14.0)	
High eye pressure			0.191
Yes	51 (89.8)	80.0 (13.0)	
No	449 (10.2)	82.7 (13.9)	
Cataract			0.121
Yes	106 (21.2)	81.0 (14.2)	
No	394 (78.8)	82.8 (13.7)	
Diabetic retinopathy			0.025
Yes	20 (4.0)	75.8 (11.8)	
No	480 (96.0)	82.7 (13.8)	
Age-related macular degeneration			0.527
Yes	10 (2.0)	80.3 (12.4)	
No	490 (98.0)	82.5 (13.8)	
Refractive error			0.520
Yes	45 (9.0)	82.3 (13.5)	
No	455 (91.0)	83.3 (16.5)	
Dry eye			0.023
Yes	152 (30.4)	80.4 (14.2)	
No	348 (69.6)	83.3 (13.5)	
Blurry vision			<0.0001
Yes	188 (37.6)	76.7 (13.8)	
No	312 (62.4)	85.9 (12.6)	
Double vision			<0.0001
Yes	39 (7.8)	70.1 (14.2)	
No	461 (92.2)	83.5 (13.3)	
Floaters			
Yes	131 (26.2)	80.4 (12.7)	0.040
No	369 (73.8)	83.2 (14.1)	

When examining which participant demographics accounted for the VFQ-9 score, transportation and insurance type/status were the only items chosen in stepwise regression and accounted for 11.4% of variance (AICC = 3,065, adjusted *R*^2^ = 0.114). For models examining the impact of self-reported eye conditions on VFQ-9, blurry vision and double vision accounted for 13% of variance of the VFQ-9 and were the eye conditions meeting criteria in stepwise regression (AICC = 3,057, adjusted *R*^2^ = 0.13). Modeling of all factors together revealed that whether one was a driver, insurance type/status, self-reported glaucoma or glaucoma suspect, self-reported blurry vision, and self-reported double vision most accounted for the VFQ-9 score. These items comprised 21.8% of the variance of the composite measure and produced the lowest AICC of the stepwise models (AICC = 3,006, adjusted *R*^2^ = 0.218).

Supplementary Table 1 presents the demographic characteristics stratified by the three FQHC clinics. Clinic 1 had a higher percentage of patients ≥60 years of age than the other two clinics. Clinics 1 and 2 had a higher percentage of African American patients than did Clinic 3. Clinic 1 had a lower percentage of patients who were employed compared to Clinics 2 and 3. Clinic 3 had a higher percentage of patients who were married or in domestic partnerships, and Clinic 2 had a higher percentage of patients who were divorced separated, or single, compared to the other clinics. Clinic 1 had a higher percentage of patients on Medicaid or Medicare than the other clinics. Clinic 2 had a higher percentage of patients with private health insurance. Clinic 3 had a higher percentage of clinics with no health insurance. Clinic 1 had a higher percentage of patients who had less than a high school education.

## 4 Discussion

The current study which focused on three FQHCs in a lower-income, rural region of Alabama found that VFQ-9 composite scores averaged 82, indicating a minor-to-moderate impairment in vision-targeted quality of life. Only one previous study examined the performance of the VFQ-9 in patients seeking at an eye screening at a FQHC located in Flint, Michigan (12). Like the current study, this FQHC is situated in a low-income community with a high percentage of African Americans, but this region is urban rather than rural as in our study. Their average VFQ-9 composite score was very similar to that reported herein, i.e., 79. The VFQ-9 has not been extensively used in the literature so comparison to other studies is challenging, especially since studies using the VFQ-9 address specific ophthalmic conditions (13, 14). During a follow-up visit in the Study on Osteoporotic Fractures (10), a comprehensive eye examination was included along with administration of the VFQ-9. The study included 5,482 women of which 88% were white persons, which is in sharp contrast to the demographics of the current study’s participants, one-third of which were men and approximately half were African American. We note that average scores in the current study with a large percentage of African Americans from lower-income, rural regions had average scores about 10 points worse than the sample from the Study on Osteoporotic Fractures. Thus, it is important to identify factors that impact VFQ-9 scores among patients from FQHCs.

Although the VFQ-9 is a vision-targeted, health-related quality of life measure, the literature on the parent VFQ-25 questionnaire indicates that non-vision-related issues impact VFQ scores (e.g., regional economy (15), health resources available in the region (15), education (16), income (17)). In the current study the most parsimonious model of VFQ-9 scores included two non-visual characteristics. Having no health insurance lowered the VFQ-9 score. In addition, being a driver and having access to a vehicle for transportation to the FQHC appointment increased the VFQ-9 score, as compared to those participants who depended on a ride from another person, took public transportation, or walked. Research indicates being a driver allows for a level of freedom and independence that is critical for social engagement and participation, and thus, it is not surprising that it contributes to quality of life (18, 19). In fact several prospective studies have shown that driving cessation is associated with incident depression (20–22) and also makes it more challenging to seek out medical care (23). Another factor which compounds the issue is the rurality of participants showing that those in nonurban areas have increased transportation challenges resulting in poorer healthcare access (24).

The final model accounting for the largest proportion of variance of the VFQ-9 given our selection criterion also included some self-reported eye conditions, namely glaucoma or glaucoma suspect, blurry vision, and double vision. Both glaucoma and glaucoma suspect status have been widely reported to be associated with reduced vision-targeted health related quality of life (25–27). Since our sampling strategy targeted patients who were at-risk for glaucoma associated disease, it is not surprising that self-reported glaucoma and glaucoma suspect were variables in the final model. The prior literature also suggests that many of the other eye conditions we asked about, particularly AMD, cataract, and diabetic retinopathy, impact quality of life; however, the current study did not show they had a significant impact VFQ-9 scores. It could be that these conditions were not severe enough to impact daily experience but this remains unknown. The limited number of persons found with AMD or diabetic retinopathy might also explain the lack of association. Patients reporting blurry vision or double vision also were part of the final model, both of which can be caused by many different etiologies.

Strengths of the current study consist of its focus on understanding vision-targeted, health-related quality of life in rural FQHCs, a unique federally-funded healthcare delivery model in the US designed for medically underserved areas and populations. Only one other study has examined vision-targeted quality of life in an FQHC, and the current and previous study are in agreement that the VFQ-9 scores are very similar, showing on average moderate impairment in quality of life. The sample size of the current study (i.e., 500) is large and targeted at people who were at risk for glaucoma. We also sought to identify demographic characteristics as well as self-reported eye conditions that predicted FQHC scores. Study limitations must also be acknowledged. Our study focused on one FQHC organization using three clinics, so generalizability is unknown. However, we are now embarking on a new telemedicine program in other FQHC clinics in Alabama, using a very similar protocol to examine generalizability. While we examined many demographic variables in terms of their impact on VFQ-9 scores, we did not include income, although persons seeking care from FQHCs typically have lower incomes. There were some demographic differences among the three clinics. We would like to emphasize that the eye conditions we studied were self-reported, not ophthalmologist diagnosed, however we are currently collecting this data in our ongoing eye screening program where the VFQ-9 is also being administered.

In summary, we have shown that vision-targeted, health-related quality of life is moderately reduced in patients seeking care at FQHCs in rural Alabama. Our program focused on patients who had risk factors for glaucoma associated disorders. The best-fitting model of predictors for VFQ-9 scores were self-reported glaucoma or glaucoma suspect, blurry vision, double vision, being a driver, and having no health insurance. The VFQ-9 is a good candidate for a vision-target health-related quality of life measure for future eye health interventions to be implemented at rural FQHCs.

## Data Availability

The raw data supporting the conclusions of this article will be made available by the authors, without undue reservation.
