# Supplementary material for: Association between self-reported eye conditions in patients at rural federally qualified health centers and vision-targeted health-related quality of life: the AL-SIGHT study
Source: Front Med (Lausanne). 2025 Jan 15;12:1498413. doi: 10.3389/fmed.2025.1498413 (PMC11774997; doi:10.3389/fmed.2025.1498413)
Supplement: Supplementary file 1 [file Table_1.DOCX]

Supplementary Table 1. Demographic characteristics of the sample stratified by the three FQHC clinics.

|  | N (%) | | |  |
| --- | --- | --- | --- | --- |
|  | Clinic 1  N = 148 | Clinic 2  N = 165 | Clinic 3  N = 186 | P-value |
|  |  |  |  |  |
| Age, years |  |  |  |  |
| 18 - 39 | 8 (5.4) | 15 (9.1) | 16 (8.6) | 0.0414 |
| 40 - 59 | 58 (39.2) | 77 (46.7) | 98 (52.7) |  |
| ≥ 60 | 82 (55.4) | 73 (44.2) | 72 (38.7) |  |
|  |  |  |  |  |
| Gender |  |  |  |  |
| Men | 53 (35.8) | 56 (33.9) | 68 (36.6) | 0.8725 |
| Women | 95 (64.2) | 109 (66.1) | 118 (63.4) |  |
|  |  |  |  |  |
| Race |  |  |  |  |
| African American | 68 (46.0) | 107 (64.9) | 53 (28.5) | <0.0001 |
| White persons | 79 (53.4) | 54 (32.7) | 124 (66.7) |  |
| Other^1^ | 1 (0.7) | 4 (2.4) | 9 (4.8) |  |
|  |  |  |  |  |
| Employment |  |  |  |  |
| Employed full- or part-time | 32 (21.6) | 50 (30.3) | 66 (35.5) | 0.0023 |
| Retired | 51 (34.5) | 43 (26.1) | 42 (22.6) |  |
| Unemployed or unable to work | 63 (42.6) | 67 (40.6) | 63 (33.9) |  |
|  |  |  |  |  |
| Marital status |  |  |  |  |
| Married or domestic partnership | 73 (49.3) | 71 (43.0) | 111 (59.7) | 0.0184 |
| Divorced, separated, or single | 54 (36.5) | 75 (45.5) | 60 (32.3) |  |
| Widowed | 21 (14.2) | 19 (11.5) | 15 (8.1) |  |
|  |  |  |  |  |
| Health insurance |  |  |  |  |
| Medicaid | 44 (29.7) | 30 (18.2) | 27 (14.5) | 0.0020 |
| Medicare | 61 (41.2) | 51 (30.9) | 48 (25.8) | 0.0104 |
| Private insurance | 55 (37.2) | 81 (49.1) | 80 (43.0) | 0.1038 |
| No insurance | 16 (10.8) | 23 (13.9) | 44 (23.7) | 0.0039 |
|  |  |  |  |  |
| Education^2^ |  |  |  |  |
| Less than high school | 49 (33.3) | 32 (19.4) | 38 (20.4) | 0.0059 |
| High school graduate or more | 98 (66.7) | 133 (80.6) | 148 (79.6) |  |
|  |  |  |  |  |
| Transportation |  |  |  |  |
| Drove themselves to screening | 103 (69.6) | 122 (73.9) | 145 (78.0) | 0.2218 |
| Someone else drove them, public  transportation, or walked^3^ | 45 (30.4) | 43 (26.0) | 41 (22.1) | 0.4991 |
|  |  |  |  |  |
